# Batch-Mask: Automated Image Segmentation for Organisms with Limbless or Non-Standard Body Forms

**DOI:** 10.1093/icb/icac036

**Published:** 2022-05-16

**Authors:** John David Curlis, Timothy Renney, Alison R Davis Rabosky, Talia Y Moore

**Affiliations:** Ecology and Evolutionary Biology and Museum of Zoology, University of Michigan, 1105 N University Ave, Michigan 48109, USA; Computer Science, University of Michigan, Michigan 48109, USA; Ecology and Evolutionary Biology and Museum of Zoology, University of Michigan, 1105 N University Ave, Michigan 48109, USA; Ecology and Evolutionary Biology and Museum of Zoology, University of Michigan, 1105 N University Ave, Michigan 48109, USA; Mechanical Engineering and Robotics Institute, University of Michigan, 2505 Hayward St, Ann Arbor, Michigan 48109, USA

## Abstract

Efficient comparisons of biological color patterns are critical for understanding the mechanisms by which organisms evolve in nature, including sexual selection, predator–prey interactions, and thermoregulation. However, limbless, elongate, or spiral-shaped organisms do not conform to the standard orientation and photographic techniques required for many automated analyses. Currently, large-scale color analysis of elongate animals requires time-consuming manual landmarking, which reduces their representation in coloration research despite their ecological importance. We present Batch-Mask: an automated, customizable workflow to automatically analyze large photographic datasets to isolate non-standard biological organisms from the background. Batch-Mask is completely open-source and does not depend on any proprietary software. We also present a user guide for fine-tuning weights to a custom dataset and incorporating existing manual visual analysis tools (e.g., micaToolbox) into a single automated workflow for comparing color patterns across images. Batch-Mask was 60x faster than manual landmarking and produced masks that correctly identified 96% of all snake pixels. To validate our approach, we used micaToolbox to compare pattern energy in a sample set of snake photographs segmented by Batch-Mask and humans and found no significant difference in the output results. The fine-tuned weights, user guide, and automated workflow substantially decrease the amount of time and attention required to quantitatively analyze non-standard biological subjects. With these tools, biologists can compare color, pattern, and shape differences in large datasets that include significant morphological variation in elongate body forms. This advance is especially valuable for comparative analyses of natural history collections across a broad range of morphologies. Through landmark-free automation, Batch-Mask can greatly expand the scale of space, time, or taxonomic breadth across which color variation can be quantitatively examined.

## Introduction

The increasing digitization of museum specimens and the convenience of digital photography provide unparalleled opportunity to quantify how color varies across the entire tree of life. However, high morphological shape variation across taxa poses a challenge for automated image analysis tools, requiring prohibitively labor-intensive analysis with manual approaches. Snakes in particular demonstrate impressive variation in coloration and patterning (Allen et al. 2013; Davis Rabosky et al. 2016) that serve critical organismal functions (e.g., anti-predator signaling (Brodie III 1993; Greene and McDiarmid 1981), thermoregulation (Clusella Trullas et al. 2007), camouflage (Isaac and Gregory 2013), and luring (Hagman et al. 2008)). Despite the iconic role of snake coloration in ecology and evolution, analysis of snakes and other elongate organisms lags behind taxa like fish, insects, and birds, specifically due to challenges in automating color pattern quantification (see these admirable, but qualitative, approaches (Farooq and Uetz 2020, 2021)).

When using photography to collect color pattern data, it is essential to identify which portions of a photograph are associated with the biological subjects and calibration tools (i.e., masking). Generally, standardizing preparation and photographing protocols reduces postural variation and enables comparison among specimens by facilitating the isolation of a biological subject. Morphological features, such as limbs and fins, are often used as landmarks to identify color variation in homologous regions (Van Belleghem et al. 2018; Schwartz and Alfaro 2021). Because snakes and many other animals lack appendages, their elongated body forms cannot be consistently positioned for photographic data collection. Snakes are usually coiled into circles or spirals for practicality (Simmons 2014), but the number, the diameter, and direction of the coils (clockwise or counterclockwise) vary greatly because snake length spans six orders of magnitude (Feldman et al. 2015). Such high disparity in morphology and posture hinders the application of traditional image processing techniques.

Recently, machine learning has facilitated the automated detection and visual categorization of biological information in large and complex datasets (Li et al. 2018; Alber et al. 2019). Machine learning can be performed by neural networks, which consist of processing nodes that distribute information to neighboring nodes (Suk 2017). These networks are trained to perform specific tasks by providing a dataset in which the task has already been performed (i.e., training set; see Glossary). Then, the trained neural network performs the same task to an unlabeled dataset (i.e., inference). By including sufficient variability in the training set, the trained neural network can robustly perform the task on diverse real-world biological data that vary in color, position, size, and resolution with applications as far-reaching as automated detection of pedestrians for self-driving cars (Bu et al. 2019), cancer from mammograms (Yassin et al. 2018), and invasive plant species in an ecosystem (Shiferaw et al. 2019). A machine learning approach has great potential for accelerating the analysis of visible phenotypes and is already being used to count reproductive structures on plants (Davis et al. 2020), measure fish abundance underwater (Ditria et al. 2020), and identify bird species (Kumar and Das 2019).

**Fig. 1:**
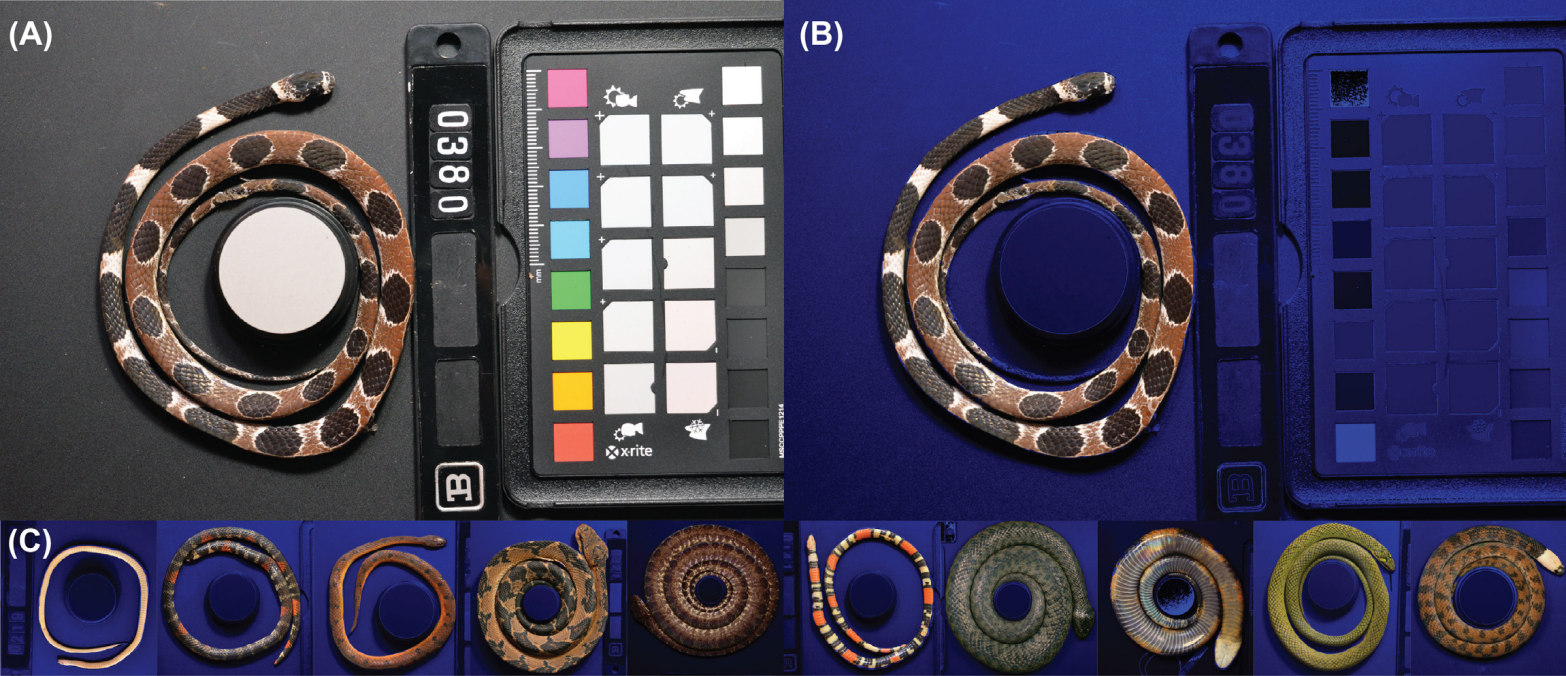
Batch-Mask  uses a neural network to take **(A)** unlabeled photographs of circular or coiled biological specimens to generate **(B)**a background-masked image. **(C)**Batch-Mask is 60x faster than manual landmarking for specimens that vary in color, pattern, thickness, orientation, and lighting.

**Fig. 2:**
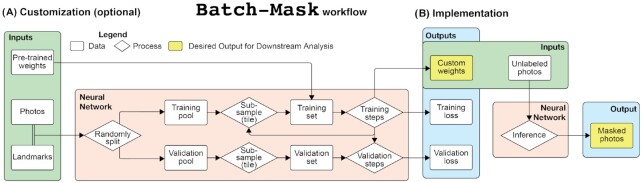
Summary diagram of the Batch-Mask workflow. **(A)** Landmarked data from a few photographs are used to train a neural network and generate fine-tuned weights. This step is unnecessary for datasets visually similar to coiled snakes. **(B)** Biological subjects are automatically isolated from an unlimited number of photographs without landmarks.

Here, we present an automated and customizable workflow (Batch-Mask) using a region-based convolutional neural network (R-CNN) to identify and isolate pixels associated with biological specimens from photographs (Fig. 1). First, we describe how we used labeled photographs to train the neural network for non-standard organisms (Fig. 2A). Then, we use the inferred weights to automate masking of unlabeled photographs (Fig. 2B). Finally, we demonstrate how Batch-Mask combines with existing manual image processing tools to automate analysis of organismal features. Due to their challenging variability in color, color pattern, size, and shape, we use a diverse benchmark photographic dataset of 33 species of neotropical snakes (UMMZ 2021) to assess our methods. Because weights are fine-tuned for a diverse dataset of coiled snakes, Batch-Mask is readily applied or trained to analyze other limbless, elongate, or coiled forms, including whole organisms, organs, or tissues. Furthermore, we include detailed instructions to adapt the neural network for identifying and isolating non-coiled biological specimens lacking appendages for reference points. The software has no dependencies on proprietary software and is freely available to download and implement using either a local desktop or cloud-based services. By using a neural network to accommodate variation in morphology and posture, this approach facilitates the automated analysis of diverse datasets for ecological and evolutionary analysis of color patterns.

## Requirements and inputs

### Requirements

The Batch-Mask source code can be run on a local machine with access to a GPU, but we present a solution that leverages Google Colab, a cloud-based service that can be any machine with internet access. Programs such as ImageJ (Schneider et al. 2012) or TPSDIG (Rohlf 2018) can be used to landmark the borders of a snake in training set photographs. A list of required Python libraries is included in the source code. Users with no computer science background have successfully implemented this tool — no specialized training or expertise is required. For biologists interested in customizing the neural network, we provide guidelines to ensure high-quality masking in the Parameter Optimization section.

### Obtaining images

We trained and tested Batch-Mask  on an open-source dataset of Neotropical snakes (UMMZ 2021). All specimens were photographed before preservation using a Nikon D7000 digital SLR camera (Nikon Inc., Melville, NY, USA) with a Coastal Optics UV-VIS-IR 60 mm F/4 macro lens (Jenoptik Optical Systems, Jupiter, FL, USA) using variable shutter speeds, F-stops, and ISO. The camera was mounted on a tripod, angled straight downward above the specimen, and positioned at a height that varied with specimen size. Rather than using the onboard camera flash, each specimen was illuminated from multiple angles using fluorescent and UV light bulbs. The background of each specimen was a blue or black PVC mat (Elviros). Each photograph contained one specimen, a color standard (X-rite Colorchecker Passport Pro), and a circular gray standard (40% Spectralon Diffuse Reflectance Standard) that was used for size calibration. Photographs were saved as JPG files, but Batch-Mask is compatible with any image file type.

To facilitate accurate color comparisons, we wrote a custom macro in Photoshop (Adobe Inc., San Jose, USA) tool to calibrate the color in each photograph. We also used the OpenCV (Bradski 2000) GaussianBlur function with a 5x5-pixel kernel size to pre-process the photographs.

### Creating labeled data

To train and implement our model, we labeled a set of 151 photographs (Set 1 in the dataset) that included species with diverse colors and patterns and both dorsal and ventral views of each snake. We recommend that training datasets include a wide range of color, size, shape, and pattern variation to maximize generalizability and minimize overfitting.

We used the tpsDig program (Rohlf 2018) to manually place landmarks along each side of the snake’s body to indicate the pixels associated with the snake. We wrote a custom script to convert the tps outline into a JSON file. Alternatively, automated image processing techniques such as watershedding or thresholding could be used to generate snake outlines, but we found these methods to be less reliable for our dataset than manually landmarking (data not shown).

Note that output masks are highly dependent on labeled data. In our dataset, lateral snake scales visible from the ventral view were excluded from ventral landmarking, due to substantially different color patterning (Supplementary Figure S1), resulting in some output masks of ventral photographs that identify regions more biologically relevant than the edge of the snake’s body.

## Training and implementation

If coiled or circular subjects are being analyzed, refer to the userguide (https://github.com/EMBiRLab/batch-mask) to begin implementation without training. Here, we describe how to train Batch-Mask to a novel ground truth dataset to facilitate customization for biological subjects that differ greatly from the visual appearance of snakes. We highly recommend starting training with the fine-tuned weights we provide because they are customized for organism segmentation. Training from the fine-tuned weights we provide, rather than pre-trained weights available for generalized image segmentation (Abdulla 2017), greatly increases mask quality and reduces the time needed to customize the neural network to other biological subjects.

### Creating training and validation sets

We randomly divided the 151 landmarked JSON files into a training pool of 135-labeled photographs and a validation pool of 16-labeled photographs, a ratio of 9:1 (see (Guyon 1997) to determine optimal ratio). In every training step, we randomly selected a photograph from the training pool, then randomly sampled one 512x512-pixel square image (tile) from the photograph for the training set. We created one fixed validation set by randomly choosing 32 x,y coordinates and sampling a 512x512-pixel tile at each location from each photograph in the validation pool. Note that no validation tiles overlap with training tiles because they are sampled from different pools of photographs. However, validation tiles may stochastically overlap with each other.

Because the output masks generated by the neural network are limited to 28x28 pixels, subdividing large images into tiles removes the need to scale down the entire image, resulting in more precise masking, while increasing computation time. This approach has the added benefit of increasing the dataset size – each portion of the animal will be represented in multiple different tiles (e.g., sometimes in the middle of a tile, sometimes at the edge), providing more context for automated identification. Thus, tiling can be considered a form of data augmentation that does not require additional manual landmarking.

### Training the neural network

Batch-Mask utilizes a customized region-based convolutional neural network (R-CNN) model (He et al. 2017) to generate masks of snakes in photographs. This neural network uses the training process to fine-tune mask weights (*W_FT_*) from pre-trained weights (*W_PT_*) provided with Mask R-CNN (obtained from training on the COCO dataset (Abdulla 2017)). On Google Colab (Abadi et al. 2015), we set the GPU count to 1 and 1 image per GPU. Our learning rate was 0.0001. All other parameters were set to the default values in the configuration file.

The number of validation steps must equal the number of tiles in the validation set, so that loss is calculated on the full validation set for every epoch. Mask R-CNN suggests using a 2:1 ratio of training to validation steps (Abdulla 2017). The number of training and validation steps in an epoch does not affect model accuracy, but if training and validation loss values converge after a single epoch, decreasing the number of training steps will reveal the progression of loss values. Decreasing training steps should be accompanied by decreasing validation steps, such that a roughly 2:1 ratio is maintained. If loss values take more than 12 h to converge, the number of training steps can be increased. If both training and validation loss plateau at non-zero values, see parameter optimization to adjust model settings.

The training resulting in the best masks used 450 training and 50 validation steps for each epoch. We trained for 20 epochs (24.2 h), each lasting 1.21 h. Training and validation losses plateaued at 16 epochs (used for inference), after which validation losses began increasing (likely due to overfitting).

### Implementation on Unlabeled Data (Inference)

To demonstrate the utility of our automated workflow to accurately process images outside of our training and validation sets, we implemented Batch-Mask on a test set of 50 unlabeled photographs (Set 2 in UMMZ 2021), each subdivided into 212 tiles of 512x512-pixel resolution with 100 pixels of overlap with neighboring tiles in each direction.

Inference required 25 min to mask 50 unlabeled images. By comparison, manually generating a JSON file of ROIs for an equal number of images would require approximately 25 h for a trained human (based on landmarking rates in the training set). Because these photographs have no landmarks, we created a Python workflow that displays a random subset of masks overlaid on original photos to qualitatively assess accuracy.

## Accuracy and validation

### Loss

**Fig. 3:**
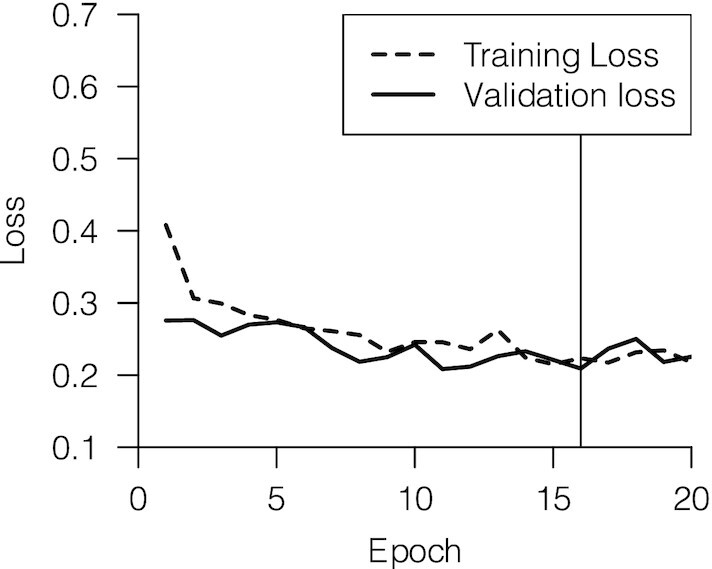
Loss per epoch for a successful training process. Training loss values decreased exponentially in the first epoch. Model weights corresponding to epoch 16, indicated by the black line, were used for inference because this point is the onset of the validation loss plateau.

We calculated training and validation losses after each epoch using the ratio of (1) pixels correctly identified as the specimen by Batch-Mask, divided by (2) all pixels identified as specimen by manual landmarking (Supplementary Table S1, Fig. 3). This loss equation does not penalize incorrectly identified pixels (i.e., background pixels misidentified by Batch-Mask as specimen pixels). Only training, not validation, loss values inform the training process.

Based on validation loss, Batch-Mask successfully and rapidly isolated pixels associated with biological subjects with up to 96% accuracy (see Supplementary Table S1) . To maximize the size of our training and validation sets, we used the validation loss at the epoch used for inference to represent the accuracy of the trained neural network, instead of inferring masks for a labeled test set.

### Accuracy for downstream color analysis

To test whether errors in machine-learned inference affected the accuracy of color analyses, we used pattern energy, computed by micaToolbox, as our metric (See ‘Integrating and automating existing tools’ section for how to incorporate micaToolbox into the Batch-Mask workflow). Pattern energy is a shape-independent metric of visual granularity computed as the standard deviation of the pixels filtered at a range of frequency bands (Troscianko and Stevens 2015). Here, we computed the pattern energy with respect to different frequency ranges in each visible color channel to determine whether losses were associated with specific colors or species.

**Fig. 4:**
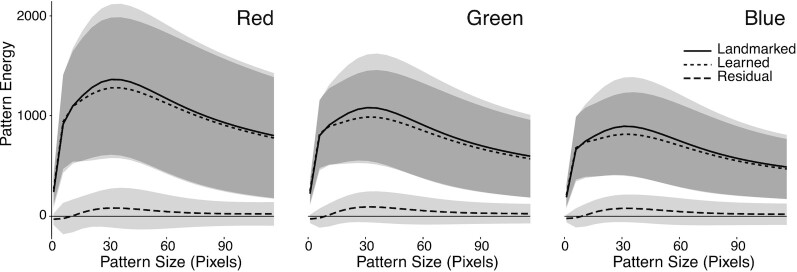
The mean residual differences (dashed line) between the pattern energies computed using the hand-labeled (solid line) and the inferred (dotted line) masks for each photograph in the training dataset. Differences are sorted by color channel: red, green, and blue. The clouds represent the standard deviation from the mean for each pattern size.

We compared two masks for each photograph in the training set: (1) hand-labeled masks and (2) masks produced by Batch-Mask after 16 epochs of training. We computed the pattern energy for each visible color channel for each photograph. Pattern energy as a function of pattern size for each color channel showed no significant differences between hand-labeled and Batch-Mask datasets (paired t-tests, all *P* < 0.05, Fig. 4). These results demonstrate that pixel-wise differences between the hand-labeled and inferred datasets do not significantly compromise the quality of downstream color analyses.

## Parameter optimization

To assist with troubleshooting and customization of the workflow, we discuss settings as they relate to loss, mask outputs, and computation time. Other settings, such as learning momentum, relative loss values, and mask shape, can also be modified but will not be discussed here.

### Troubleshooting and modifying settings

The three parameters that have the most effect on output are learning rate, tile size (resolution), and tile overlap. Learning rate controls the magnitude of the correction to the weights in response to a mismatch between training output and the labeled data. High learning rate values cause the training and validation loss values to diverge or wildly oscillate, whereas small values result in slower convergence but less oscillation. We recommend starting with smaller learning rate values and slowly increasing between training sessions to improve performance. Note, the Mask R-CNN code includes by default a learning rate decay throughout a training session, which was not modified for this method.

**Fig. 5:**
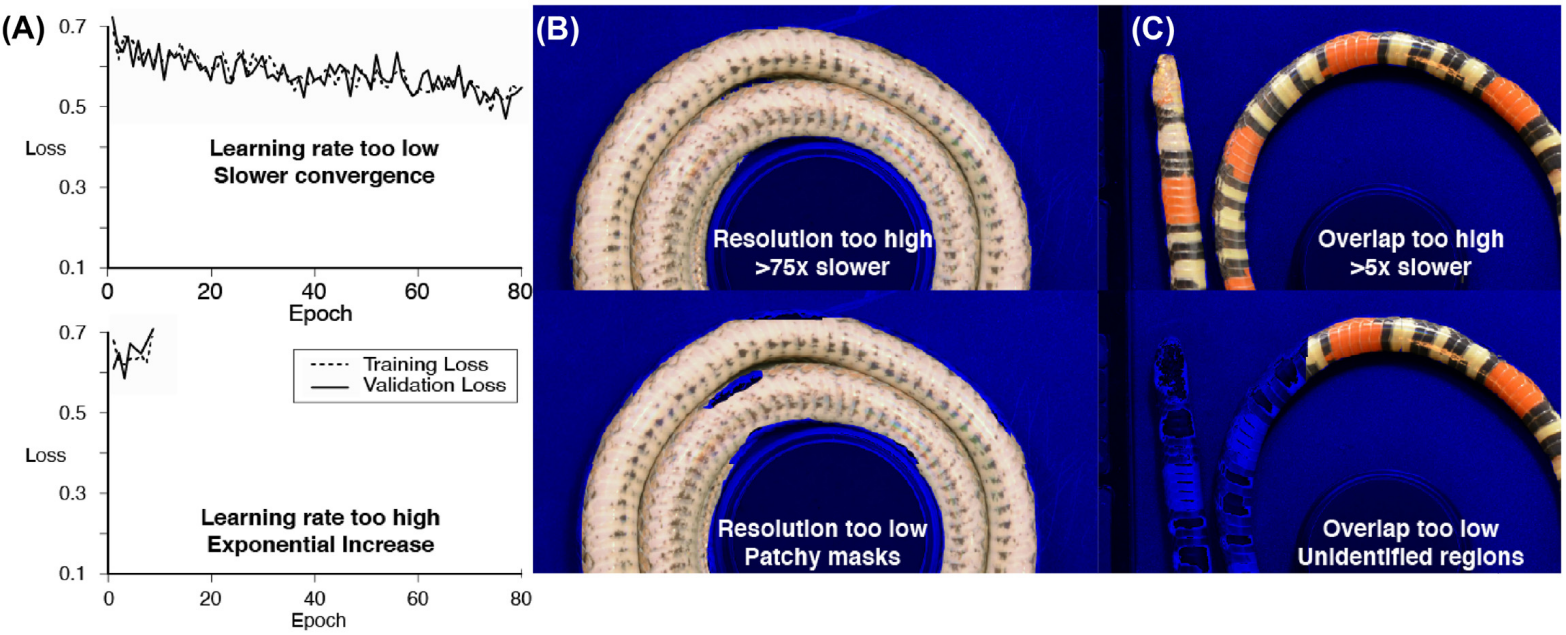
Visual guide to troubleshooting the training process. Loss plots inform **(A)** Learning rate. Mask quality informs **(B)** Subdivision/Resolution and/or **(C)** Overlap. The top row output indicates that the parameter should be increased. The bottom row output indicates that the parameter should be decreased. Note that the loss plots are exaggerated to show the most recognizable patterns and were not generated by training results.

The resolution of each tile, number of subdivisions, and overlap between neighboring tiles sampled from each photograph are interdependent. If ROI portions are unidentified (Fig. 5A, top) or if undesired regions are included, decreasing tile size (increasing the number of subdivisions for the same amount of overlap) increases mask resolution. A higher resolution typically produces a more accurate model but requires exponentially more memory (Fig. 5A, bottom).

If mask inaccuracies correspond to the edges of subdivided image tiles, we recommend increasing overlap (the number of pixels shared between neighboring tiles). With higher overlap, features are viewed in multiple contexts, providing more opportunities for proper identification. Alternatively, decreasing overlap reduces computation time for acceptable output masks.

### Overfitting

Accuracy is highest when the distribution of variation in training and validation sets match. Overfitting occurs when the model is complex enough to memorize the entire training dataset, causing poor generalization (training loss decreases, but validation loss plateaus or increases after a plateau). To avoid overfitting, expand the size of, and variation within, the training set (specimen size, shape, pattern diversity, color) to more accurately reflect the variation in the validation set. If diversity cannot be expanded with additional images, randomly changing the brightness and the hue of each image introduces useful variation in the training set.

### Post-processing

Because loss calculation does not penalize non-ROI pixels misidentified as ROI pixels, the resulting inferred masks will likely include pixels outside of the snake. To eliminate these outliers, our script uses the OpenCV function findContours to identify the largest contiguous unmasked area and eliminate unconnected areas. This step is helpful if portions of the background are recognized incorrectly as ROI. If the ROI is in more than one contiguous piece, this function can be changed to recognize two or more unmasked areas.

### Customization considerations

Future customization could include incorporating fully convolutional functionality to the neural network to identify multiple classes of objects or multiple objects of the same class. This would enable the simultaneous identification of snakes and calibration objects or multiple snakes in the same picture, respectively. However, the user should be aware that this would require labelling all the objects and assigning them to the different classes in the training set. Previous studies have successfully used human data labelling services to rapidly construct such training sets (Russell et al. 2008).

## Integrating and automating existing tools

A key advantage of Batch-Mask  is the ease of incorporating existing color analysis tools into the Batch-Mask Python workflow to automate the analysis of large datasets. Any python-compatible image processing software can be incorporated into the Batch-Mask workflow. This effectively automates analyses that previously required multiple manual inputs per photograph. The open-access code we provide (https://github.com/EMBiRLab/batch-mask) demonstrates this process by incorporating the existing micaToolbox (Troscianko and Stevens 2015) plugin for ImageJ (Schneider et al. 2012) into a fully automated Python workflow.

### Automated color analysis workflow

We incorporated micaToolbox (Troscianko and Stevens 2015) functions into the Batch-Mask workflow to combine color channels, scale the image by size, and create a single MSPEC file for pattern energy analysis of complexity in the snake ROI. To automate the process of generating MSPEC images, we modified the micaToolbox script to load the ROIs directly from the JSON file and batch-generate MSPEC files for multiple specimens at once.

To calibrate the size in each photograph automatically, we wrote a script to identify a circular gray color standard in each photograph using the Circle Hough Transform algorithm (Bradski 2000). The gray standard and snake ROIs were combined and exported into a single JSON file.

## Implications

Batch-Mask can be used to facilitate the rapid, automated identification and isolation of complex biological forms in photographs, enabling efficient quantification of phenotypic variation. Although this masking is but a single component of any color pattern analysis workflow, it can easily become the rate-limiting step for organisms with limbless, “non-standard” body forms that can vary across so many spatial axes. Moreover, some of the most powerful color pattern analysis tools, such as patternize, do not currently include masking capabilities and assume that if the user wants to analyze organisms isolated from their backgrounds, the user must complete the masking step beforehand (Van Belleghem et al. 2018). Other color pattern analysis tools, such as micaToolbox, require manual landmarking as part of the base workflow (Troscianko and Stevens 2015).

Masking is especially important in automated species recognition to prevent computer vision algorithms from using potentially irrelevant background information (Ribeiro et al. 2016). Batch-Mask is therefore a useful tool to pre-process images for automated species identification algorithms (see description in Durso et al. 2021).

While obtaining digital photographs to capture phenotypic diversity among organisms has become increasingly easy, methods for analyzing such datasets often rely on the user to identify, landmark, and/or mask organisms by hand before the data can be fully utilized. Advances in machine learning have addressed this by substantially reducing the time and effort required to analyze larger datasets, yet the sheer complexity and diversity of biological forms continue to present challenges for generalized methods. The limbless, slender body shapes that are characteristic of snakes are often problematic for neural networks because many cannot feasibly be arranged in a straight line and must be coiled to fit inside the frame of a photograph. Batch-Mask can also be easily trained to identify elongate forms in non-coiled postures (Supplementary Figure S2) , which enables broader implementation of elongate body forms exhibiting *in vivo* behaviors and shape configurations. Elongate phenotypes are quite prevalent across taxa, including organisms such as other reptiles (e.g., amphisbaenians and other lizards), amphibians (e.g., tadpoles, sirens, caecilians), fishes (e.g., eels, hagfishes), annelids (e.g., polychaetes, earthworms, leeches), gastropods (e.g, slugs, snails, nudibranchs), myriapods (e.g., centipedes, millipedes), and flatworms. Such shapes are also found in homologous appendages like limbs, tails, and tentacles, as well as organs and tissues such as intestines and sperm. Given the pervasiveness of these complex forms, the Batch-Mask workflow has extremely far-reaching applications including fields from ecology and evolutionary biology to human medicine.

## Funding

The work was funded by the University of Michigan through startup funds and an MCubed grant to TYM and ARDR and through the Undergraduate Research Opportunity Program to TR. Publication costs were supported by the University of Michigan Museum of Zoology to ARDR.

## Glossary

Mask R-CNN: A Region-based Convolutional Neural Network that uses a mix of convolutional and fully connected layers to classify images.
Mask: Binary array with the same dimensions as the image, with 1 indicating snake pixels and 0 non-snake pixels.
Region of interest (ROI): Parts of an image outlined by a polygon or designated by a binary array.
Label: Human-generated ROIs for the training and validation sets.
Landmarking: Identifying the locations of comparable morphological features among distinct biological specimens.
JSON: JavaScript Object Notation formatted file comprising landmark and image information.
Weights: Values applied and changed during training to fit the neural network to data. Pre-trained weights (*W_PT_*) were provided with the standard Mask R-CNN model trained on the COCO dataset. Fine-tuned weights (*W_FT_*) fit during training are utilized, but not changed, during inference.
Training pool: Set of 135 labeled photographs from Set 1 from which sample tiles are extracted to generate the training set. There is no overlap between training and validation pools.
Training set: Set of tiles sampled from photographs in the training pool. There is no overlap between training and validation sets.
Training step: The neural network predicts labels for tiles from the training set, then updates the model weights if the model fails to match the true mask.
Validation pool: Set of 16 photographs from Set 1 from which sample tiles are extracted to generate the validation set.
Validation set: Set of labeled tiles used for the validation steps of the training process.
Validation step: The neural network predicts labels for images from the validation set but does not change model weights. Validation steps calculate loss values on a labeled dataset distinct from the training set. This allows detection of overfitting to the training dataset.
Loss: Accuracy of model predictions for each pixel calculated by the number of pixels shared by the label and the mask divided by the number of pixels in the label only (He et al. 2017).
Epoch: A set number of training and validation steps. Model weights are saved after each epoch. Changing the number of training and validation steps per epoch changes how frequently model accuracy is assessed.
Training Process: Using several epochs of training and validation to fine-tune model weights.
Inference Process: Using fine-tuned weights to generate masks for data outside of training and validation sets. Note that weights are not updated during inference.

## Supplementary Material

icac036_Supplemental_File

## Data Availability

Output masks: https://doi.org/10.7302/3xwv-7n71. Code: https://github.com/EMBiRLab/batch-mask.

## Notes

Authors contributed equally.

From the symposium “Open source solutions in experimental design’’ presented at the annual meeting of the Society for Integrative and Comparative Biology virtual annual meeting, January 3–February 28, 2022.
