# Supplementary material for: Batch-Mask: Automated Image Segmentation for Organisms with Limbless or Non-Standard Body Forms
Source: Integr Comp Biol. 2022 May 16;62(4):1111–20. doi: 10.1093/icb/icac036 (PMC9617216; doi:10.1093/icb/icac036)
Supplement: icac036_Supplemental_File [file icac036_supplemental_file.zip › icb-2022-0022-File002.pdf]

Appendix

Dorsal and ventral variability

The accuracy for the ventral photographs was lower than for dorsal photographs, likely due to variation in the original posture and lateral scale overlap in the ventral view (Table S1, Figure S1). In general, the landmarks on the dorsal view of a snake aligned perfectly with the edge of the snake's body, as there is typically no noticeable distinction between dorsal and lateral scales. This discrepancy caused the model to more often misidentify lateral scales in the ventral photographs, in turn resulting in lower accuracy. This discrepancy demonstrates that model accuracy greatly relies on original landmarking, and model accuracy increases when target features have clear visual differences from the background.

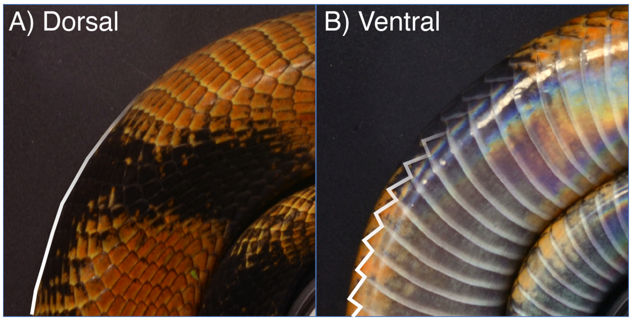

Fig. S1. Comparison between A) dorsal photograph of a snake and B) ventral photograph of the same snake. White outlines show landmarking borders. Note that ventral and lateral scales are less visually distinct from each other than the background is from most portions of the snake.

Table S1. Model accuracy using different combinations of training and validation images. Dorsal and ventral photographs were used to create different datasets (see Figure S1). Accuracy is calculated in two ways. Intersection/Union (I/U) by the number of shared pixels between the labeled data and the identified ROI (Intersection) divided by the total area identified by both the labels and the ROI (Union), which accounts for both Type I and Type II error. Intersection/Labeled (I/L) is calculated by intersection over Labeled pixels (equivalent to the accuracy metric in [He et al., 2017]), which determines how many of the labeled pixels were accurately inferred, but does not penalize pixels incorrectly identified as ROI. The I/L value is used to train the model. Variability in the ventral landmarked data contributed to lower accuracy when these data were incorporated into the validation set. The identity of training and validation photos were consistent across experimental conditions. A mask trained on dorsal and ventral data was used for inference in Section 3.3.

Training set	Validation set	I/U	I/L
Dorsal	Dorsal	88.1%	96.0%
	Ventral	74.1%	93.9%
	Dorsal + Ventral	80.2%	94.8%
Ventral	Dorsal	40.8%	43.1%
	Ventral	73.0%	81.5%
	Dorsal + Ventral	58.9%	64.7%
Dorsal + Ventral	Dorsal	87.6%	95.3%
	Ventral	80.8%	91.2%
	Dorsal + Ventral	83.4%	93.0%

Customization for other datasets

Here we describe `Batch-Mask` training results for a dataset that is visually distinct from the photographs used in the training and validation sets we provide. The images are frames from high-speed videos of live snake anti-predator behaviors [Moore et al., 2020]. These snakes take on a variety of body postures and orientations, all of which are dissimilar to the coiled forms used for preservation. Although some individual snakes in the behavior dataset may be included in the training or validation sets, the background, lighting conditions, posture, and camera are all distinct. With the fine-tuned weights, the neural network was trained with 100 epochs, with 630 training steps and 60 validation steps per epoch (Figure S2 B). The loss reached a minimum of 0.1058 and resulted in highly accurate masks. These results demonstrate that the fine-tuned weights we provide result in more accurate output masks even for biological subjects that are not spiral-shaped or coiled.

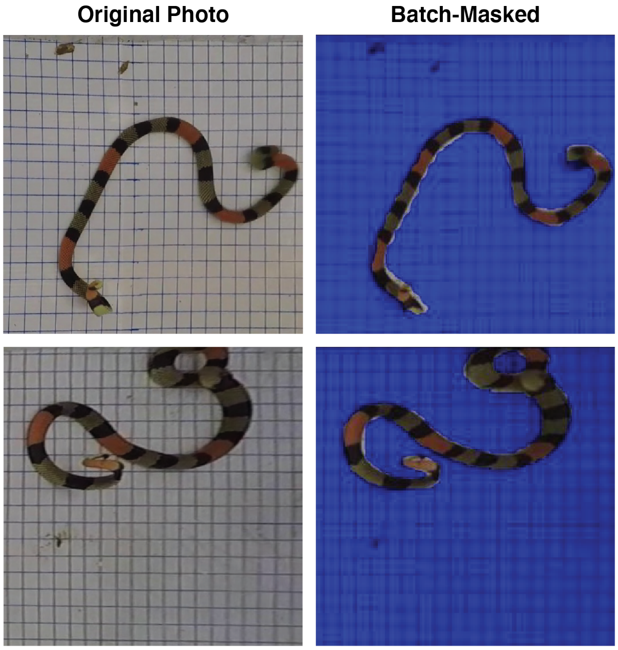

Fig. S2. Masks generated by training the `Batch-Mask` workflow using videos of live snakes performing anti-predator behaviors.
