# Supplementary material for: Batch-Mask: Automated Image Segmentation for Organisms with Limbless or Non-Standard Body Forms
Source: Integr Comp Biol. 2022 May 16;62(4):1111–20. doi: 10.1093/icb/icac036 (PMC9617216; doi:10.1093/icb/icac036)
Supplement: icac036_Supplemental_File [file icac036_supplemental_file.zip › icb-2022-0022-File003.zip!/Final 00 ICB-2022-0022.R2/BWFig4.pdf]

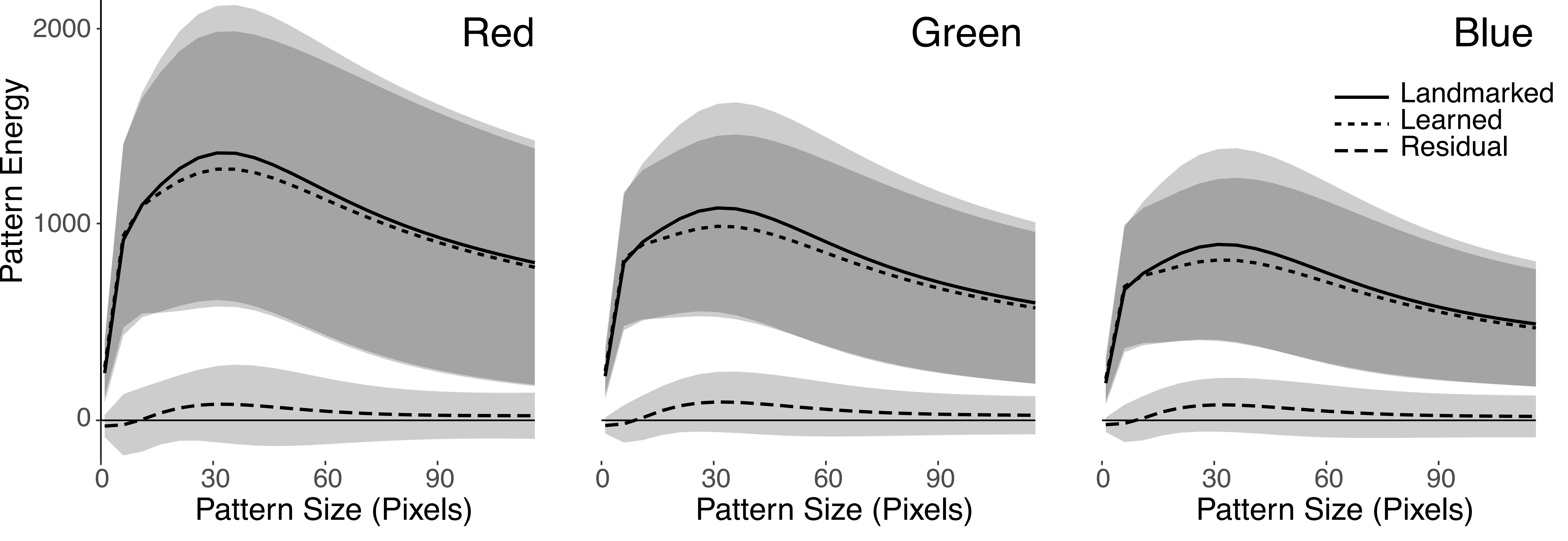

Red
Green
Blue
Landmarked
Learned
Residual
Pattern Energy
2000
1000
0
0
30
60
90
Pattern Size (Pixels)
