# Supplementary material for: Batch-Mask: Automated Image Segmentation for Organisms with Limbless or Non-Standard Body Forms
Source: Integr Comp Biol. 2022 May 16;62(4):1111–20. doi: 10.1093/icb/icac036 (PMC9617216; doi:10.1093/icb/icac036)
Supplement: icac036_Supplemental_File [file icac036_supplemental_file.zip › icb-2022-0022-File003.zip!/Final 00 ICB-2022-0022.R2/BWFig3.pdf]

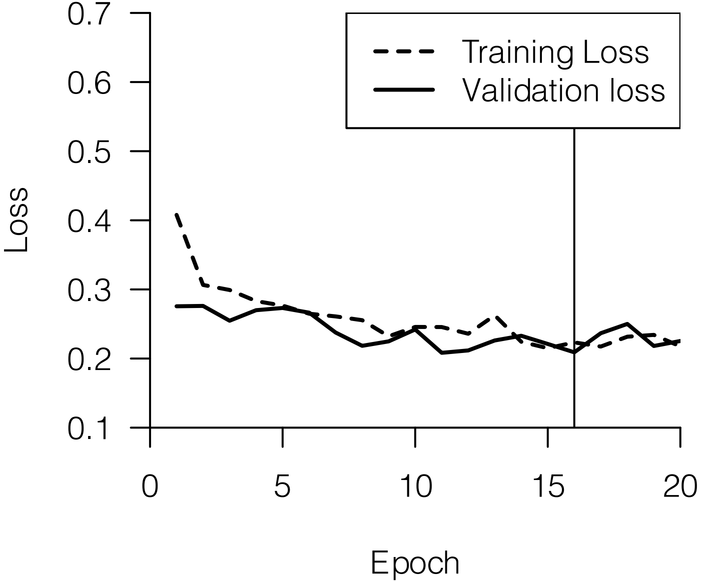

Training Loss
Validation loss
Loss
Epoch
0.1
0.2
0.3
0.4
0.5
0.6
0.7
0
5
10
15
20
