# Supplementary material for: Batch-Mask: Automated Image Segmentation for Organisms with Limbless or Non-Standard Body Forms
Source: Integr Comp Biol. 2022 May 16;62(4):1111–20. doi: 10.1093/icb/icac036 (PMC9617216; doi:10.1093/icb/icac036)
Supplement: icac036_Supplemental_File [file icac036_supplemental_file.zip › icb-2022-0022-File003.zip!/Final 00 ICB-2022-0022.R2/ColorFig2.pdf]

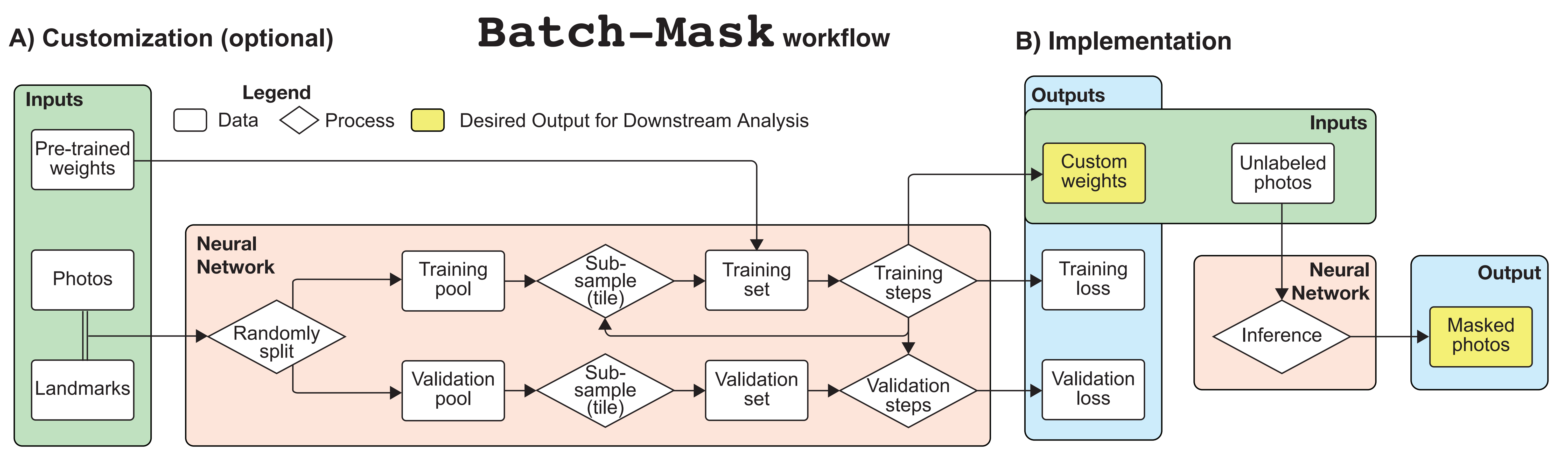
A) Customization (optional)
Batch-Mask workflow
B) Implementation
Inputs
Legend
Data
Process
Desired Output for Downstream Analysis
Pre-trained weights
Photos
Landmarks
Neural Network
Randomly split
Training pool
Sub-sample (tile)
Training set
Training steps
Validation pool
Sub-sample (tile)
Validation set
Validation steps
Outputs
Inputs
Custom weights
Unlabeled photos
Training loss
Validation loss
Neural Network
Inference
Output
Masked photos
