# Supplementary material for: Batch-Mask: Automated Image Segmentation for Organisms with Limbless or Non-Standard Body Forms
Source: Integr Comp Biol. 2022 May 16;62(4):1111–20. doi: 10.1093/icb/icac036 (PMC9617216; doi:10.1093/icb/icac036)
Supplement: icac036_Supplemental_File [file icac036_supplemental_file.zip › icb-2022-0022-File003.zip!/Final 00 ICB-2022-0022.R2/ColorFig5.pdf]

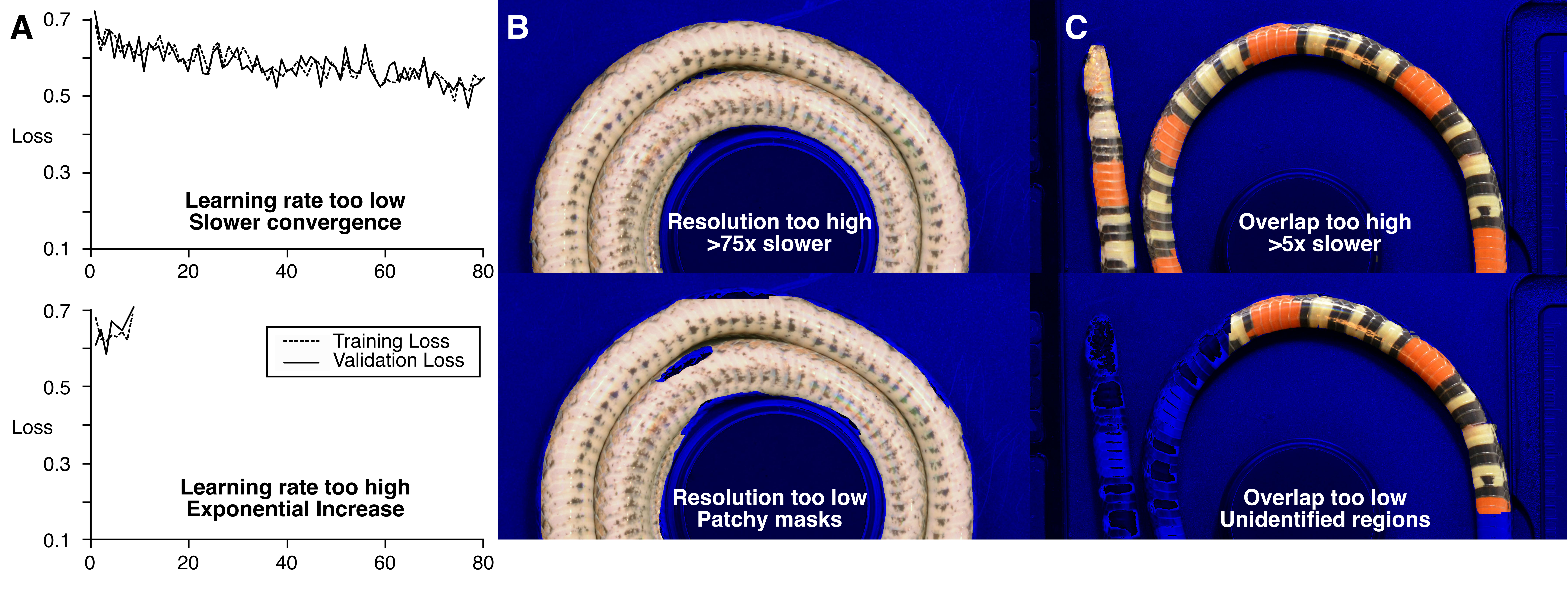

A
0.7
0.5
Loss
0.3
0.1
0
20
40
60
80
Learning rate too low
Slower convergence
Training Loss
Validation Loss
Learning rate too high
Exponential Increase
B
Resolution too high
>75x slower
Resolution too low
Patchy masks
C
Overlap too high
>5x slower
Overlap too low
Unidentified regions
